# An Effect of a Carbon-Containing Additive in the Structure of a Friction Material on Temperature of the Wet Clutch Disc

**DOI:** 10.3390/ma15020464

**Published:** 2022-01-08

**Authors:** Aleksander Yevtushenko, Piotr Grzes, Aleksander Ilyushenko, Andrey Liashok

**Affiliations:** 1 Department of Mechanics and Applied Computer Science, Faculty of Mechanical Engineering, Bialystok University of Technology (BUT), 45C Wiejska Street, 15-351 Bialystok, Poland; a.yevtushenko@pb.edu.pl; 2 The State Scientific Institution “Powder Metallurgy Institute” (SSI PMI), National Academy of Sciences of Belarus, 41 Platonova Street, 220005 Minsk, Belarus; Alexil@mail.belpak.by (A.I.); sdilav@tut.by (A.L.)

**Keywords:** friction material, clutch, temperature, frictional heating, numerical model

## Abstract

This paper consists of two parts. The first one contains a description and methodology of the composite material used as friction material in clutches. Four variants of such material, differing in the type of carbon additive (the elemental graphite, pencil graphite and foundry coke powder of various fractions) were considered. Thermal conductivity, thermal diffusivity as well as the specific heat all materials were determined experimentally. On the inertial IM-58 stand, a simulation of the braking process of the friction pair consisting of a steel disc with friction material and a counterpart in the form of a homogeneous steel disc was carried out. On this basis, averaged coefficients of friction, unchanging in the entire sliding process, were found for the four friction pairs. The experimental data obtained in the first stage were used in the second stage to develop two (2D and 3D) numerical models of the friction heating process of the friction pairs under consideration. For four variants of the friction material, a comparative spatial-temporal temperature analysis was performed using both models. It was found that a simplified axisymmetric (2D) model can be used to estimate the maximum temperature with high accuracy. The lowest maximum temperature (115.6 °C) obtained for the same total friction work was achieved on the friction surface of the material with the addition of GP-1.

## 1. Introduction

The friction materials are widely used in friction pairs of vehicles, tractors, motorcycles, machine tools and others. Such are the hydro mechanical gearboxes, the oil-cooled brakes, the clutches and etc. [1,2,3]. From the reliable operation of such units and mechanisms depends on the safety of both service personnel and surrounding people [4].

The main advantages of friction materials are high efficiency in torque transmission, low wear [5], smoothness and transparency of operation [4], absence of seizure and formation of burns [6], effective dissipation of thermal energy generated during a slip [7], possibility to minimize the force of switching on and switching off the friction device due to creation of the optimum kinematic scheme.

Known friction materials containing fibers [8], are based on a polymer matrix [9], however, for heavy-loaded friction systems, the most common are powder sintered friction materials (PSFM) [10]. The PSFMs are produced by powder metallurgy and have a complex composite structure that combines metal and non-metal components. As the basis of the friction material, iron and copper have become the most widespread. An iron is used in friction pairs without lubrication. For friction pairs with lubrication, copper-based materials are the most common. Copper allows for efficient material processing, heat dissipation, and low wear [11,12]. However, pure copper does not provide the required values of the coefficient of friction and wear intensity. To change these properties, graphite powder is used. For these purposes natural graphite powders with a size of 80 μm, and finely dispersed, with a size of 8–10 μm can be used [13]. Coarse graphite provides a significantly higher coefficient of friction. It has been shown that foundry coke powder can be used as an additive in the PSFM composition [14]. Its presence makes it possible to increase significantly the value of the coefficient of friction of the material [14].

A feature of the operation of friction materials is slipping, accompanied by both an increase and a decrease in pressure for a short time period, while the velocity can either decrease in case of a friction pair closure, and increase—when it is opened. Short process durations and high velocities lead to the release of a large amount of heat and hence temperature. This causes the destruction of the surface layer, determining wear of the material and, consequently, the service life of the entire machine or mechanism [15]. The studies on the stability, corrosion-resistance, and applicability of ternary mixed metal oxides/metallates (tungstates) and the new additive-manufactured hybrid high entropy alloys were carried out in the articles [16,17].

The development of PSFMs requires the manufacture of samples, carrying out both full-scale and bench tests. The duration of such a period can be either several months or several years. In the case of PSFMs, solving the problem of heat dissipation during frictional sliding by computational methods, would significantly reduce the development time of the friction material for given operating conditions. Modeling the process of temperature change in the friction zone, depending on the geometrical dimensions of the friction disc and steel disc, the amount of lubricant supplied, as well as the composition of the PSFM itself, would allow to predict its operation at a preliminary stage.

The transient temperature fields and quasi-static stresses of the components of a wet multi-plate clutch during a typical engaging process were determined numerically in one of the pioneering works [18]. The analyzed friction pair consisted of alternately arranged steel discs and discs with a steel core lined with layers of friction material. In the calculation model of the friction clutch, the directionality of the thermophysical properties of materials (steel and sintered bronze) was taken into account. The temperature fields were determined using the finite difference method, while the thermal stresses at selected time moments of the friction process by the finite element method. The convective heat transfer coefficient applied in the computational model of the multidisc clutch was in the range 130–1890 W m^−2^ K^−1^. A computer simulation of the friction process taking into account the thermomechanical contact of the working elements of the multidisc clutch with thermoelastic instability effect was carried out in the article [19]. An attempt to identify the features of the hot spots generation process in a wet multi-disc clutch was made in the paper [20]. The results obtained on the basis of the thermoelastic contact model were compared with the corresponding experimental data.

Computational models of friction clutches were discussed in terms of their transmissibility and engaging control strategy, and the influence of temperature, velocity, contact pressure and wear on the generated torque was analyzed [21].

Significant progress has been observed in the development of the analytical models of the heat generation process due to friction in friction components of clutches [22]. In this paper, apart from the calculations using an exact solution to the thermal problem of friction related to the heating of the dry clutch, the results of the relevant bench tests with the use of a thermal imaging camera are also presented. Analytical differential and integral wear was developed to prepare detailed maps of changes in friction material wear during clutch operation [23]. The proposed approach also allows for the analysis of changes with the braking time of the torque transmitted by the clutch. Two different (with a priori given heat partition coefficient or conditions of perfect thermal contact of friction) analytical models for determining the temperature during the operation of the clutch were proposed [24]. The temperature values obtained with the use of both theoretical models are in good agreement with the analogous results found with the finite element (FE) axisymmetric (2D) model. The above-mentioned analytical models that allow to determine not only the temperature, but also thermal stresses at a specific point on the working surface and inside the friction material were developed [25].

However, the greatest advances in temperature modeling of friction systems such as disc brakes and clutches stem from the use of numerical methods. Without listing all publications on this subject in detail, one should refer the reader to relevant reviews [26,27,28,29]. The studies that were not included in these articles cover the 2D FE computational model, implemented in ANSYS software [30]. It was assumed that both working elements of the clutch are heated separately with heat flux proportional to the friction power density. The ratio of heat distribution in the form of the Charron’s formula was used as the proportionality coefficient. Single and multiple clutch operation modes were considered.

The subject of temperature is closely related to other aspects of clutch operation, in particular, such as strength of materials and mechanical properties. The analysis of the temperature field and its gradients was performed during a single engaging of the carbon fabric wet clutch in order to predict damage to the composite material [31]. The latter were defined as the increase in surface roughness and the occurrence of cracks as observed on scanning electron microscopy (SEM) micrographs.

A review and evaluation of materials used for friction elements of clutches was presented in relation to specific design solutions developed from the end of the 19th century [32]. The design aspects of the complex hybrid mechanical material system of a woven fiber yarn (glass fiber with aromatic polyamide, copper, and poly-acrylic-nitrile) reinforced friction material was performed with the intention of creating separated component groups (matrix and fiber groups). It was noted that the obtained data can be used as input parameters for the development of thermomechanical models of clutches.

The purpose of this article was:

(1) development of effective 2D and 3D computational models with the use FEM to determine the temperature of the friction clutch;
(2) carrying out a comparative analysis of the temperature fields obtained by means of both models with the same friction work done;
(3) investigating the effect of a carbon-containing additive in the structure of the new four friction materials on the temperature mode of the clutch.
(4) When developing the computational models of frictional heating of the clutch, the authors used their previous experience, gained earlier in the modeling of the temperature mode of disc brakes [33,34].

## 2. Friction Materials

The basis of the composite friction material was bronze obtained from copper powder and 10% tin (BrO_10_), which contained 20% of the additive in the form of elemental graphite GE-1, pencil graphite GP-1 and foundry coke powder of various fractions C-1 and C-2.

**Figure 1:**
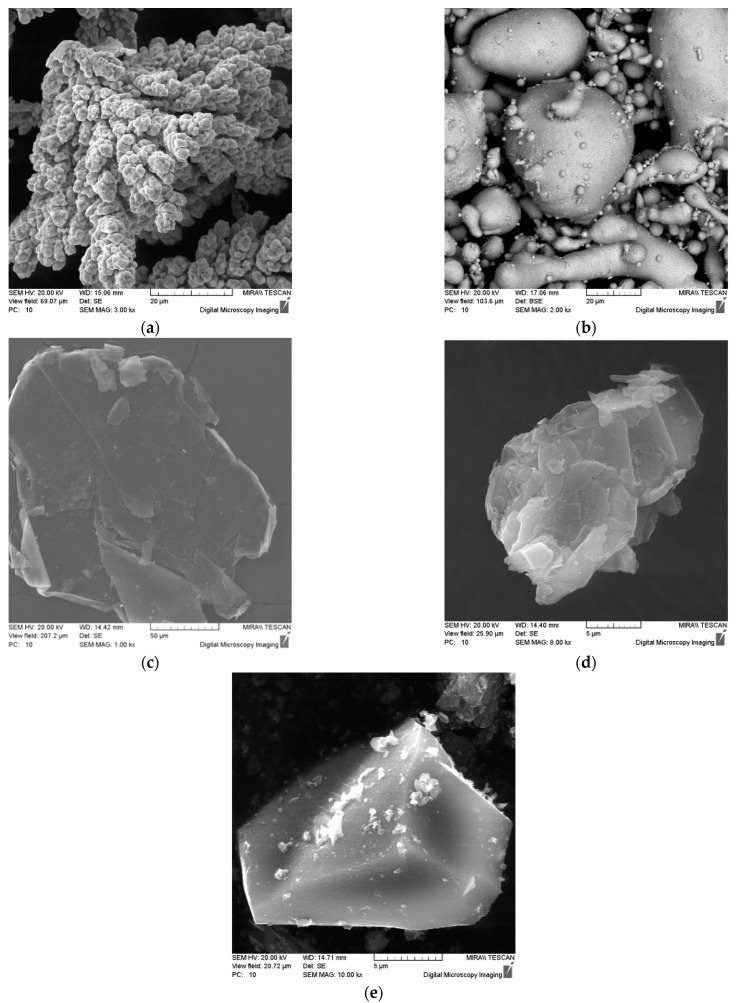
Shape of powder particles: (**a**) PMS-1; (**b**) PO-1; (**c**) GE-1; (**d**) GP-1; (**e**) C-1, C-2.

The charge of the material was obtained by mixing the initial copper powders of the PMS-1 grade with an average particle size of 80 μm (Figure 1a), tin of the PO-1 grade with an average particle size of 30 μm (Figure 1b), graphite of the element grade GE-1 with an average particle size of 100 μm (Figure 1c), pencil graphite GP-1 with an average particle size of 8 μm (Figure 1d), foundry coke powder with a size of less than 60 μm (C-1) (Figure 1e) and 160–200 μm (C-2). Mixing of the initial powders was carried out in a mixer of the “drunken” barrel type for 50 min.

It should be noted that the tribotechnical properties of the friction material are largely influenced by the amount and size of the used powder additives, which determine the contact area during friction. In addition, structure has an impact. Graphite of any grade has a layered crystalline structure, while coke is amorphous.

The used coke powder additive is obtained by crushing large pieces of coke, which is used as fuel, the cost of which is not high. Graphites of grades GE-1 and GP-1 are natural, the cost of which is 20–30% higher than the cost of foundry coke.

## 3. Experiment

**Figure 2:**
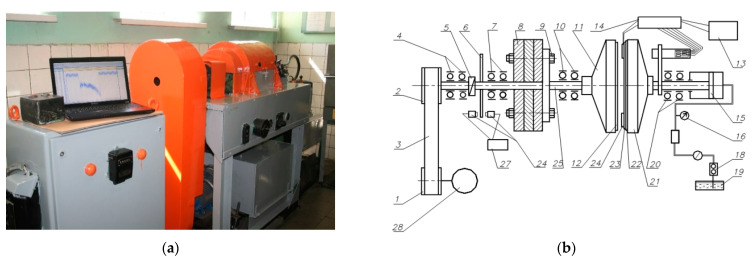
Inertial stand IM-58: (**a**) physical appearance; (**b**) structural scheme.

The essence of the test method was to simulate the braking process on the inertial stand IM-58 and record the dependence of the friction moment on the velocity and time of braking. The scheme of the IM-58 stand is shown in Figure 2.

The tests were carried out with friction by the ends of a rotating counterpart 12 and a stationary test specimen 24, which are mounted in the mandrels 11, 22 and 23 (Figure 2b). The axes of their rotation are located horizontally and mounted on bearings 4, 7, 10, 20. The rotating mandrel 11 is fixed on the spindle 25, which rotates from the electric motor 28 through the pulleys 1 and 2 and the V-belt transmission 3. The stationary mandrel 21 is fixed on the piston rod 15, mounted on two radial bearings and having the ability to rotate around its axis. The mandrel 21 and the rod 15 are held against rotation by a strain gauge beam. The load on the samples was created by a gear oil pump 18 powered from the oil tank 19 through the filter 17. The working oil pressure was regulated by the throttle and recorded by the pressure gauge 16. Fixation of readings of number of turns was carried out through the intermediate disk 6 and the speed sensor 27. The inertial masses 8 necessary to obtain the required kinetic energy are attached to the spindle flange 25 using bolts 9 and 26. After reaching the selected rotation velocity, the spindle 25 with the inertial masses 8 was disconnected from the electric motor using an overrunning cam clutch 5 and the stationary sample 24 was pressed with the required force against the rotating counterpart 12. With the electric motor turned off, the unique energy accumulator was the rotating inertial masses. Under the action of frictional forces between the counterpart and the test sample, the drive shaft along with the inertial masses gradually stopped, transmitting the torque to the amplifier 14 and personal computer 13.

**Figure 3:**
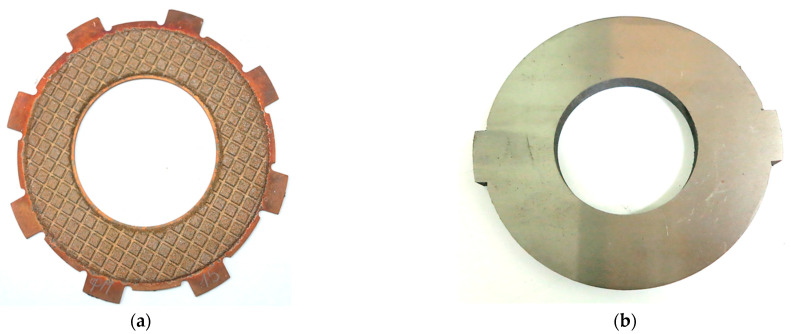
Appearance of test samples: (**a**) the friction disc; (**b**) the steel counterpart.

The appearance of the test specimens is shown in Figure 3. The friction disc was a steel base in thickness 2.5 mm, made of 65G steel on which a layer of friction material with a thickness 0.5 mm was attached. The outer and inner diameters of the friction pad were 95 mm and 65 mm, respectively. The counterpart samples used for research were made of 65G steel (0.62–0.7% C, 0.17–0.37% Si, ≤0.9–1.2% Mn, ≤0.035% P, ≤0.035% S, ≤0.25% Cr, ≤0.2% Cu) with the thickness of 10.0 mm, the Brinell hardness of 180–200 MPa and the roughness of the surface of friction Ra = 0.7–0.8 μm. Hydraulic oil of grade A (TU 38.1011282-89) intended for operation in torque converters and automatic transmissions was used as a coolant. Oil was supplied from the inner part of the disc at a rate of 1.01 min^−1^.

The results of the influence of the type of carbon-containing additive in the composition of the friction material on the thermal properties and the coefficient of friction are shown in Table 1. The coefficient of friction was recorded after 300 cycles of each test, i.e., in a steady state wear mode.

**Figure 4:**
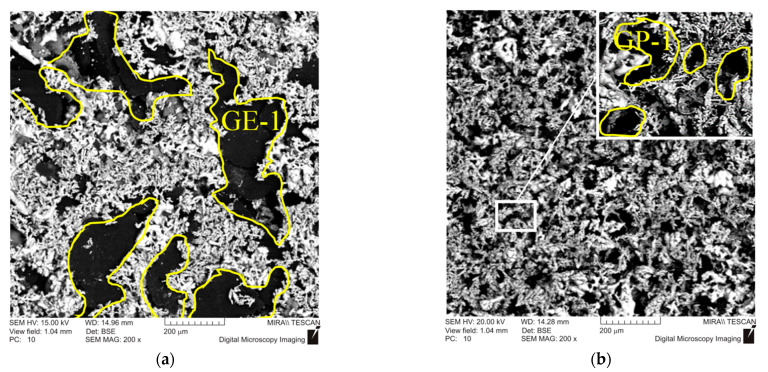
Structure of a frictional material with various mark of graphite: (**a**) GE-1; (**b**) GP-1.

The obtained data revealed that the highest value of the thermal conductivity of $44\;W\;m^{-1}\;K^{-1}$ was obtained for friction material GE-1, containing coarse graphite. In the case of fine graphite GK-1, the thermal conductivity was only $28\;W\;m^{-1}\;K^{-1}$. This may be due to the fact that the area of the metal contact of the formed tin bronze, in the case of GE-1, is significantly larger than that of GP-1 (Figure 4a). The fine powder of graphite GP-1 in the process of mixing is able to be located both between copper particles and inside them, leading to a decrease in the area of the metal contact (Figure 4b).

An even greater decrease in the thermal conductivity of the friction materials C-1 and C-2 containing foundry coke powder may be due to the significantly lower value of the thermal conductivity of coke in comparison with graphite. So in the monographs [35,36] it is shown that the thermal conductivity of coke powder of various fractions is 0.249–0.542 W m^−1^ K^−1^, while after graphitization is equal to 0.58–1.34 W m^−1^ K^−1^. For example, the thermal conductivity of graphite determined by the spatial orientation of the layers can be 233 W m^−1^ K^−1^, while pure copper is equal to 400 W m^−1^ K^−1^ [37].

The highest value 0.06 of the friction coefficient was obtained for the friction material C-2 with the addition of foundry coke powder, while for graphite’s GE-1 and GP-1 0.45 and 0.35, respectively. This may be due to both the structure of the crystal lattice and spatial orientation. Thus, graphite’s GE-1 and GP-1 have a hexagonal crystal lattice, while coke is amorphous.

## 4. Numerical Model

Numerical simulation of the friction heating process clutch during a single deceleration from the initial speed to the stop was performed on the inertial stand of the IM-58 (Figure 2). The analyzed clutch consists of two working elements: a steel disc with friction material applied to one face, and a steel counterpart (Figure 3). The calculations were performed for four (no. 1, 2, 3, 4) variants of the friction material with thermophysical properties and friction coefficients from Table 1. The values of the other input parameters are shown in Table 2. The given values of contact pressure, velocity, moments of inertia are characterized as parameters of heavily loaded friction units, clutches, mechanisms of automotive vehicles, special purpose vehicles, devices, machine tools.

**Figure 5:**
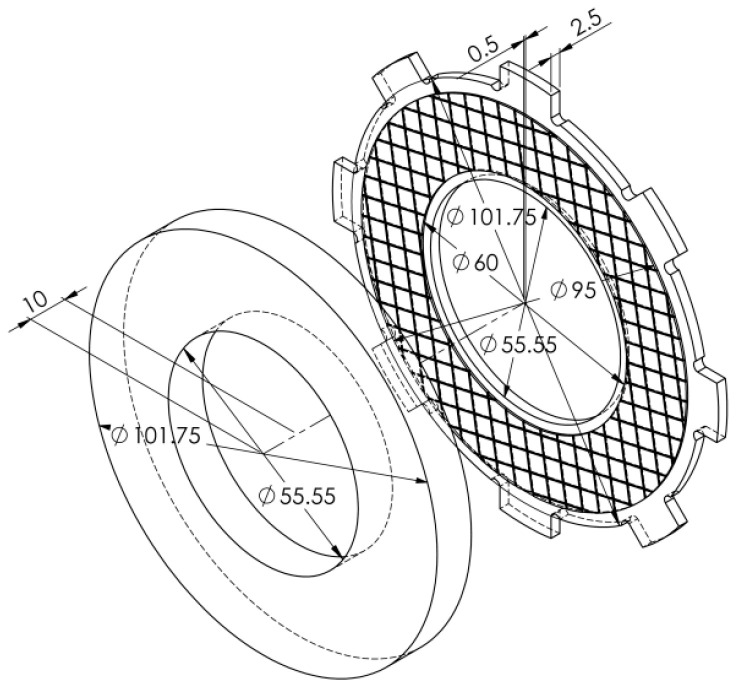
Geometrical 3D CAD model of the clutch.

The most important dimensions of both elements are shown in Figure 5.

**Figure 6:**
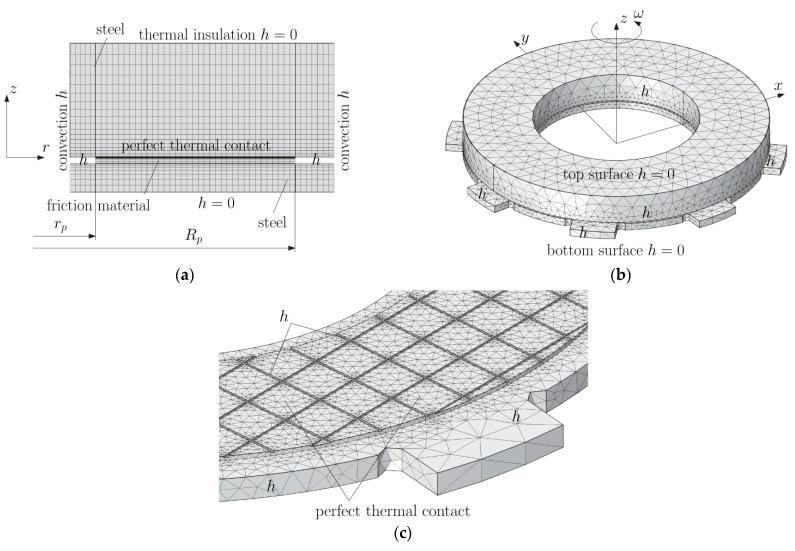
FE meshes of the clutch: (**a**) axisymmetric (2D) and (**b**,**c**) spatial (3D).

Two numerical models were developed using FEM: axisymmetric (2D) and spatial (3D). The differences in these models were in the material of the lining and the disc connected to it, which had fasteners on the outer cylindrical surface limiting its rotation. In the case of the lining from the 3D model, cuts (grooves) were taken into. In the 2D model, these cuts were not included due to axial symmetry. Thus, nominal contact surfaces in 2D and 3D models were different. The final FE (quadratic Lagrange elements) meshes created for both models are shown in Figure 6, and the corresponding numbers of elements are included in Table 3.

Taking into account the data contained in Table 2, the nominal contact surface area $A_{a}$ in the 2D model, determined by the values of the inner $r_{p}$ and outer $R_{p}$ radius of the disc containing the friction material, is equal to:

(1) $$A_{a}^{\left(2D\right)}=\pi (R_{p}^{2}-r_{p}^{2})=4261\cdot 10^{-6}\;m^{2},$$

and the clamping force P of the clutch elements during braking was:

(2) $$P=p^{\left(2D\right)}A_{a}^{\left(2D\right)}=17.04\;\mathrm{kN}.$$

Because the friction surface area in the 3D model was smaller than in the 2D model, the contact pressure was higher. On the basis of the dimensions (Figure 5), an appropriate spatial CAD geometric model of two elements of the friction pair was developed and the friction surface area was determined $A_{a}^{\left(3D\right)}=3759\cdot 10^{-6}\;m^{2}$. Then, taking into account the Formula (2), the contact pressure for the 3D model was determined:

(3) $$p^{\left(3D\right)}=\frac{P}{A_{a}^{\left(3D\right)}}=4.53\;\mathrm{MPa}.$$

With the constant contact force P and the friction coefficients unchanged in the braking process $f_{i}$ (Table 1), the reduction of the angular velocity ω in time t from the initial value $\omega_{0}$ to zero in the moments of stopping $t=t_{s,i}$ is linear:

(4) $$\omega (t)=\omega_{0}\omega^{*}(t),\;\omega^{*}(t)=1-\frac{t}{t_{s,i}},\;0\leq t\leq t_{s,i},$$

(5) $$t_{s,i}=\frac{\omega_{0}I_{0}}{f_{i}P\;r_{eq}},\;i=1,2,3,4,$$

where the equivalent radius $r_{eq}$ was calculated from the formula [33]:

(6) $$r_{eq}=\frac{2(R_{p}^{3}-r_{p}^{3})}{3(R_{p}^{2}-r_{p}^{2})}=0.0394\;m$$

**Figure 7:**
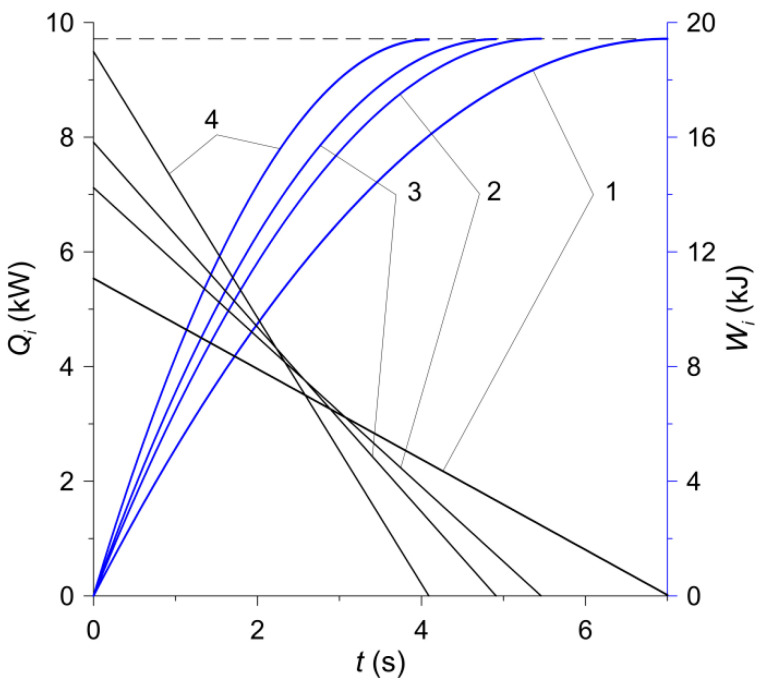
Time profiles of the power $Q_{i}$ and the work $W_{i}$, i=1, 2, 3, 4 of friction during braking.

Taking into account the Formulas (3) and (6) and the values of the coefficients of friction from the Formula (5) contained in Table 1, the next stopping times were determined for each of the four variants of the friction material: $t_{s,1}=7.02\;s$, $t_{s,2}=5.46\;s$, $t_{s,3}=4.91\;s$ and $t_{s,4}=4.09\;s$. Due to the constant contact force of the discs P (2) in both calculation models, also the stopping times for 2D and 3D models with the use of the corresponding friction materials were the same. The change in the process of braking power $Q_{i}$ and work of friction $W_{i}$, i=1,2,3,4:

(7) $$Q_{i}(t)=Q_{0,i}\omega^{*}(t),\;0\leq t\leq t_{s,i},\;Q_{0,i}=f_{i}P\omega_{0}\;r_{eq},$$

(8) $$W_{i}(t)=\int_{0}^{t}\;Q_{i}(\tau )d\tau =W_{0,i}W_{i}^{*}(t),\;W_{0,i}=Q_{0,i}t_{s,i},$$

(9) $$W_{i}^{*}(t)=\frac{t}{t_{s,i}}\left(1-\frac{t}{2t_{s,i}}\right),0\leq t\leq t_{s,i},$$

are shown in Figure 7. Based on the Formulas (8) and (9), it was established that in the moments of stopping $t=t_{s,i}$, the friction work for all four calculation variants was the same and amounted to: $W_{s,i}\equiv W_{i}(t_{s,i})=0.5W_{0,i}=19.43\;\mathrm{kJ},\;i=1,2,3,4.$ This value is equal to the initial kinetic energy of the system, calculated from the formula:

(10) $$W_{0}=0.5I_{0}\omega_{0}^{2}=19.43\;\mathrm{kJ}.$$

The simulated process concerned frictional heating during braking in time $0\leq t\leq t_{s,i}$, which made it possible to stop the rotating masses at a given moment of inertia, and then cooling the friction system during $t_{s,i}\leq t\leq 90\;s$, i=1, 2, 3, 4. During the slip of the clutch elements resulting in generation of heat in the time interval $0\leq t\leq t_{s,i}$, in the contact area, the thermal contact of friction was perfect, i.e., the sum of heat fluxes directed perpendicularly from the contact surface of the friction material and the steel disc to the inside was equal to the power density of the frictional forces, and the temperature values of the opposite surfaces were the same. At $t>t_{s,i}$, i=1, 2, 3, 4 the friction surfaces of the elements were adiabatic. Throughout the analyzed process (0≤t≤90 s), convection cooling on the friction-free surfaces with the constant heat transfer coefficient took place. The exception was the two outer frontal surfaces of the sliding elements, which were thermally insulated (h=0) both during heating and cooling (Figure 6).

In accordance with the experimental tests carried out, the relative rotational movement of the clutch components was simulated. The calculations were conducted in COMSOL Multiphysics^®^ software by using the Heat Transfer Module [38]. Additionally, in order to determine the friction work done $W_{i}$ (Figure 7), special tools, namely Global ODEs (ordinary differential equations) and DAEs (differential-algebraic equations) from Mathematics module were incorporated.

## 5. Numerical Analysis

The numerical simulation concerned the comparative analysis of the temperature fields in four friction pairs (no. 1, 2, 3, 4) obtained on the basis of the axisymmetric (2D) and spatial (3D) numerical models.

**Figure 8:**
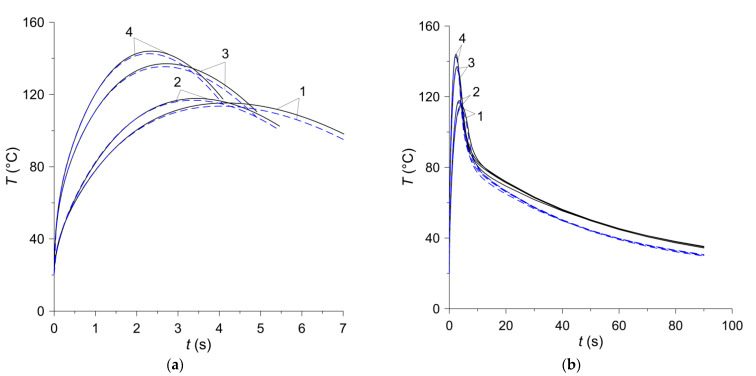
Temperature evolutions for four frictional material on the contact surface z=0 at equivalent radius $r=r_{eq}$: (**a**) during braking; (**b**) during braking and subsequent cooling. Solid curves—2D model; dashed curves—3D model.

The temperature changes on the friction surface of the disc are shown in Figure 8. The assumptions made in the analysis regarding the constant contact pressure and the linearly decreasing angular velocity of the rotating elements lead to a typical change in the temperature of the contact surface of the friction clutch elements—its value increases at the beginning of the process to the maximum value within approximately 2–4 s and then decreases slightly until it stops (Figure 8a). The differences in the time courses of temperature on the friction surface, obtained by means of two FE models during braking, are insignificant and occur only at the stage of cooling, after stopping (Figure 8b). The difference of the maximum temperature values obtained at the braking stage with the use of both calculation models for all friction materials does not exceed 2% (Table 4). In the cooling phase after stopping, the temperature of the friction surface of the disc drops gradually. The temperature values at a fixed point in time obtained by means of the 2D or 3D model for all four friction materials differ negligibly. It is noticeable that the temperature found when the axisymmetric model is each time higher than that obtained with the use of the spatial model.

**Figure 9:**
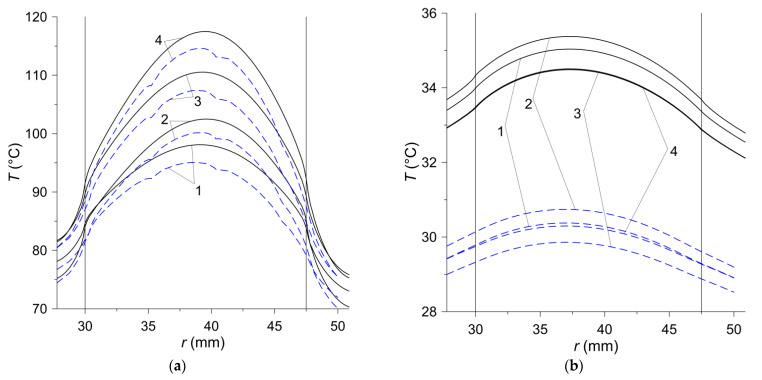
Temperature distributions for four materials along the radial variable r on the contact surface z=0 in: (**a**) stop time moments $t=t_{s,i}$, i=1, 2, 3, 4; (**b**) last moment of time $t_{end}=90\;\;s$. Solid curves—2D model; dashed curves—3D model.

The temperature distributions on the friction surface in the radial direction are presented in Figure 9. In the case of the axisymmetric model, when the temperature does not depend on the angular variable, the choice of the radial straight line along which the calculations were performed was arbitrary, while when using the 3D model, a line along the axis x was chosen (Figure 6b). In the contact area {30 mm≤r≤47.5 mm, z=0}, marked by vertical solid lines in Figure 9, the temperature distribution is shaped by a directly proportional linear dependence of the friction power density on the radial variable r (Figure 9a). The linear temperature rise in the contact area along the radius is the best seen in the initial braking stage at t=0.5 s. Outside the area of heating, a clear drop in temperature is visible. The lack of heating after stopping results in a gradual leveling of temperature (Figure 9b). The highest temperature is achieved in the area of heating in the vicinity of the equivalent radius, which, according to Formula (6), is equal to $r_{eq}=39.4\;\mathrm{mm}$. The difference between the maximum temperature values obtained with the 2D and 3D models is about 3 °C. Over time, the differences in temperature values obtained with both models increase and at the stopping time they are about 5 °C. At the time of the stop, the maximum temperature of 117.5 °C (2D model) was reached for the material no. 4 on the radius r=39.5 mm close to the equivalent radius $r_{eq}=39.4\;\mathrm{mm}$ (Figure 9a). At the end of the cooling process of the clutch friction elements, the above-mentioned temperature differences are more noticeable, but they relate to a relatively much lower temperature than during the braking stage. At the time t=90 s, the most heated material was that denoted no. 2, and the least—material no. 3, but the difference between the temperatures of these materials was insignificant (≈0.9 °C).

**Figure 10:**
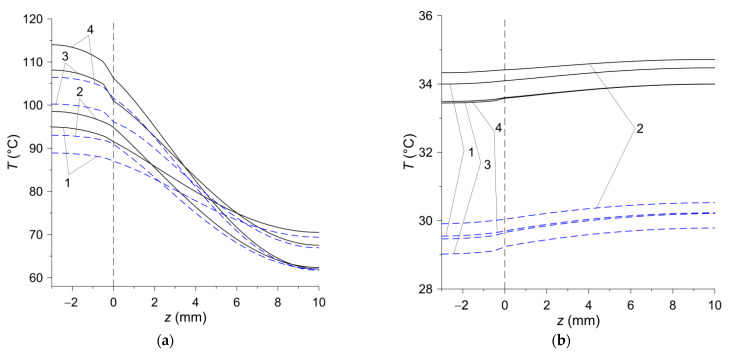
Temperature distributions for four materials in axial direction r at radius r=45  mm in: (**a**) stop time moments $t=t_{s,i}$, i=1, 2, 3, 4; (**b**) last moment of time $t_{end}=90\;\;s$. Solid curves—2D model; dashed curves—3D model.

Temperature changes in the axial z direction for a stationary disc with the friction material (−3 mm≤z≤0) and a rotating steel disc (counterpart 0≤z≤10 mm) are shown in Figure 10. In the stop times $t=t_{s,i}$, i=1, 2, 3, 4 the calculation results obtained on the basis of 2D and 3D models agree well (Figure 10a). The temperature of the stationary steel disc decreases with the distance from the contact surface z=0. The slight increase in temperature at a deflection from the contact surface in the stationary disc is associated with the known lagging effect—the decrease in temperature inside the body is slower than on its heated surface. At the end of the process $t=t_{end}$ of unforced convection cooling of the clutch after stopping, the temperature distribution along the thickness of the friction pair elements is even (Figure 10b). However, as in the radial direction (Figure 9b), the differences between the temperature values obtained with the two calculation models are noticeable (≈4 °C), but the temperature itself at that moment is not high.

**Figure 11:**
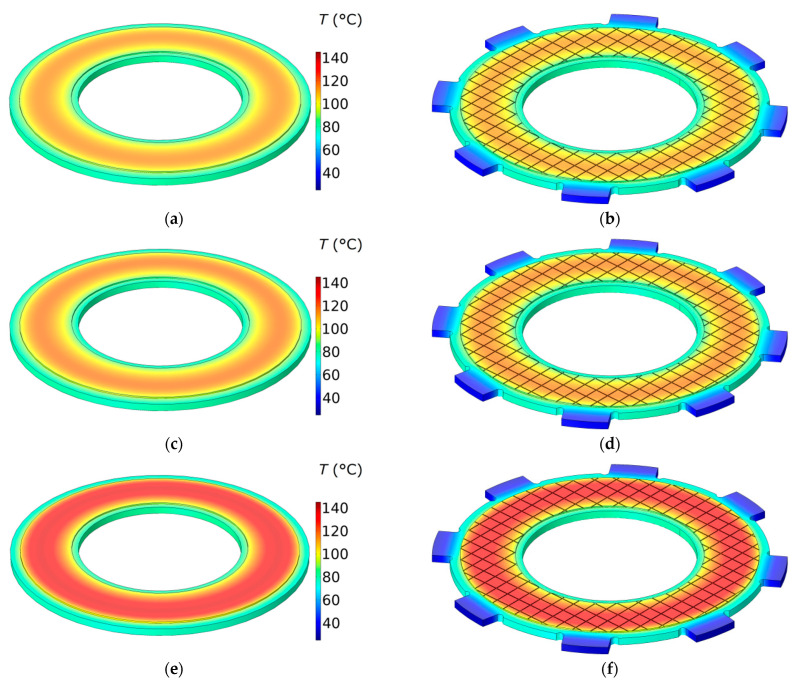

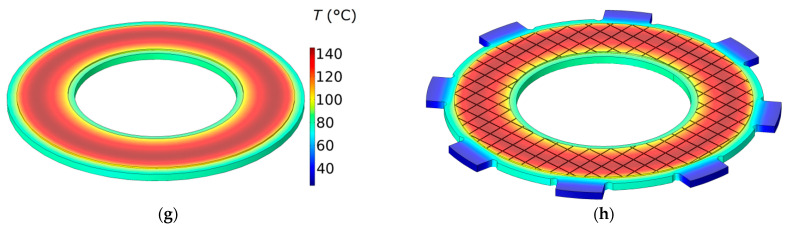
Temperature distributions on the surface of friction at the time $t_{\max,i}$, i=1, 2, 3, 4 of reaching the maximum temperature for four frictional materials: (**a**,**b**) $t_{\max,1}=4.16\;\;s$; (**c**,**d**) $t_{\max,2}=3.45\;\;s$; (**e**,**f**) $t_{\max,3}=2.72\;\;s$; (**g**,**h**) $t_{\max,4}=2.35\;\;s$. Model 2D—(**a**,**c**,**e**,**g**); model 3D—(**b**,**d**,**f**,**h**).

The temperature distributions on the friction surface obtained by means of the 2D and 3D models at the time moments $t_{\max,i}$, i=1, 2, 3, 4; when the maximum values of temperature $T_{\max}$ were reached (Table 4) are shown in Figure 11. They show the differences between the results obtained with the use of two calculation models on one hand, and on the other they show the explicitly temperature effect of using different friction materials. For the same total friction work, the temperature of the friction surface increases with the braking time reduction ($t_{s,1}=7.02\;s$, $t_{s,2}=5.46\;s$, $t_{s,3}=4.91\;s$, $t_{s,4}=4.09\;s$). The location of the area with the increased temperature in the vicinity of the equivalent radius is visible $r_{eq}$ (6).

**Figure 12:**
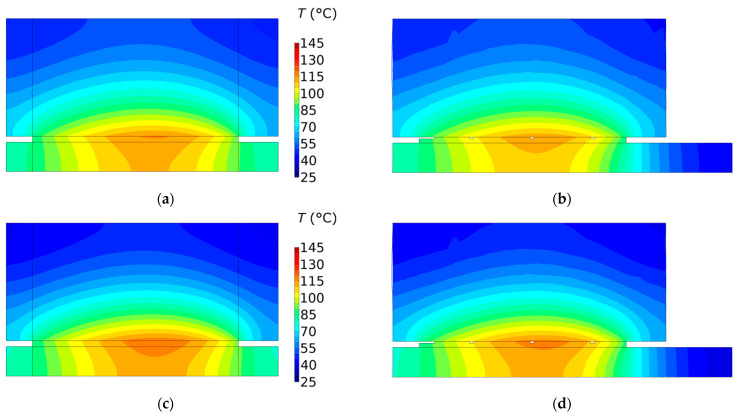

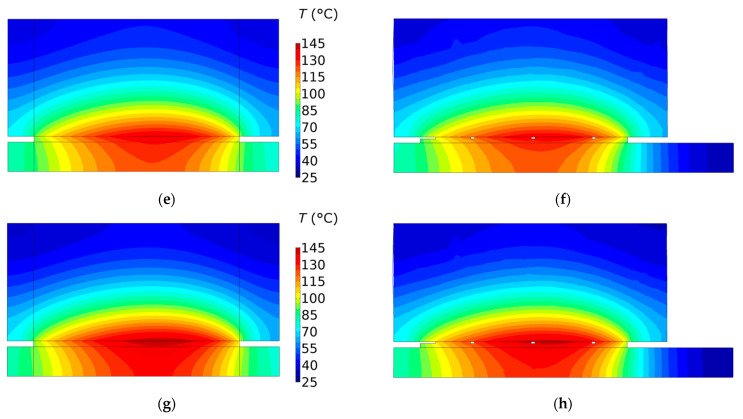
Isotherms in a plane rz at the time $t_{\max,i}$, i=1, 2, 3, 4 of reaching the maximum temperature for four frictional materials: (**a**,**b**) $t_{\max,1}=4.16\;\;s$; (**c**,**d**) $t_{\max,2}=3.45\;\;s$; (**e**,**f**) $t_{\max,3}=2.72\;\;s$; (**g**,**h**) $t_{\max,4}=2.35\;\;s$. Model 2D—(**a**,**c**,**e**,**g**); model 3D—(**b**,**d**,**f**,**h**).

The isotherms in the cross-section of the clutch in the plane rz at the moments of reaching the maximum temperature in each of the four friction materials are shown in Figure 12. The highest value of the maximum temperature $T_{\max}=145.1\;{}^{\circ}C$ was observed in the case of the material no. 4, and the lowest $T_{\max}=115.6\;{}^{\circ}C$ was determined when using the material no. 1. The maximum temperature was reached on the radii of 40.4 mm, 40.7 mm, 40.75 mm, 40.85 mm, for the material no. 1, 2, 3, 4 respectively. It can be seen that the disc with the friction material is heated in the entire thickness of 3 mm, while the counterpart is heated only to about half of its thickness (≈5mm).

## 6. Conclusions

This paper presents the results of experimental studies on the thermal and physical properties and the coefficient of friction as well as numerical simulations of the temperature mode of the new composite friction material used in the clutches. The data obtained for the four variants (no. 1, 2, 3, 4) of such material, differing in the type of carbon additive, were analyzed. The axisymmetric (2D) and the spatial (3D) numerical models of the wet clutch in order to determine the transient temperature fields of the friction discs during a single engagement and after the stop were developed. Based on the obtained results, it was found that:

(1) the highest thermal conductivity and specific heat has the material no. 2, while the lowest values of the quantities has the material no. 3;
(2) the highest coefficient of friction appears for the steel disc combined with the friction material no. 4, and the lowest for steel disc and friction material no. 1;
(3) the estimation of the maximum clutch temperature can be carried out with sufficient accuracy using a 2D model, which allows for the reduction of labor losses at the preparatory stage and computational time. The single simulation case carried out on the workstation with CPU Intel^®^ Xeon^®^ E5-2698 v4 @ 2.20GHz; RAM 64 GB (DDR4) lasted approximately 40 s, and 2200 s, when using 2D and 3D models, respectively. On the other hand, the determination of the temperature field in the elements of the friction clutch is better to carry out with the use of the 3D model;
(4) At the same total friction work done during a single clutch engagement, the lowest temperature was achieved when using friction material no. 1, and the highest for material no. 4. Also, the use of these materials resulted in the longest and shortest periods of frictional sliding, respectively.

It should be noted that in the proposed numerical models with the use of FEM, the averaged, experimentally obtained, thermophysical properties of the composite material were used. The development of the computational models that take into account the structure of the composite and the properties of individual components belongs to the future.

Subsequent studies will aimed at using the developed 2D and 3D models for assessing the effect of additives of powders (ceramic, metallic, intermetallic compounds), their particle size, distribution and the amount in the composition of the friction material on the thermal effects during friction. One of the important aspects would be to include realistic properties of a coolant. This will make it possible to assess the efficiency of their use without expensive studies on the full scale inertial test bench.

## Figures and Tables

**Table 1:** Influence of the type of carbon-containing additive on thermophysical properties and coefficient of friction.

No.	Additive	Thermal Conductivity $W\;m^{-1}\;K^{-1}$	Specific Heat $J\;{\mathrm{kg}}^{-1}\;K^{-1}$	Mass Density $\mathrm{kg}\;m^{-3}$	Thermal Diffusivity ${\mathrm{mm}}^{2}\;s^{-1}$	Specific Heat Capacity $\mathrm{MJ}\;m^{-3}\;K^{-1}$	Coefficient of Friction Dimensionless
1	GP-1	28.1	1189.2	6059	3.9	7.2	0.035
2	GE-1	44	2514.9	5832	3	15	0.045
3	C-1	13.6	438.3	5171	6	2.3	0.05
4	C-2	18.8	701.2	5362	5	3.7	0.06

**Table 2:** Operating parameters from experimental tests.

Parameter	Value
contact pressure, $p_{0}^{\left(2D\right)}$ MPa	4
initial angular velocity, $\omega_{0}$ $\mathrm{rad}\;s^{-1}$	235.6
braking torque of the rotating masses, $I_{0}$ $\mathrm{kg}\;m^{2}$	0.7
inner radius of the friction path, $r_{p}$ mm	30
outer radius of the friction path, $R_{p}$ mm	47.5
initial/ambient temperature, $T_{a}$ °C	20
heat transfer coefficient, h $W\;m^{-2}\;K^{-1}$	600

**Table 3:** Numbers of the 2D and 3D finite elements of the clutch models.

Type of Quadratic Lagrange Elements	Friction Material	Steel Plate	Steel Disc	Assembly
quadrilateral elements	175	376	940	1491
tetrahedral elements	69,014	69,946	129,267	268,227

**Table 4:** Maximum temperature obtained using the 2D and 3D FE clutch models.

Model	$T_{\max}$	$T_{\max}$	$T_{\max}$	$T_{\max}$
	1	2	3	4
2D	115.6 °C	118.4 °C	137.9 °C	145.1 °C
3D	113.7 °C	117.2 °C	136.1 °C	143.5 °C

## Data Availability

Not applicable.

## Nomenclature

$A_{a}^{\left(2D\right)}$	nominal contact surface area in the 2D model ($m^{2}$)
$A_{a}^{\left(3D\right)}$	nominal contact surface area in the 3D model ($m^{2}$)
$f_{i}$	coefficient of friction of the i−th=1,2,3,4 friction material (dimensionless)
$I_{0}$	braking torque of the rotating masses ($\mathrm{kg}\;m^{2}$)
h	heat transfer coefficient ($W\;m^{-2}K^{-1}$)
$p_{0}^{\left(2D\right)}$	contact pressure in the 2D model (MPa)
$p^{\left(3D\right)}$	contact pressure in the 3D model (MPa)
P	clamping force of the clutch elements (N)
$Q_{i}$	friction power of the i−th=1,2,3,4 friction material (W)
r	radial coordinate (m)
$r_{p},\;R_{p}$	inner and ouer radius of the friction path, respectively (m)
$r_{eq}$	equivalent radius of the contact region (m)
t	time (s)
$t_{end}$	total time of the process equal to 90 s (s)
$t_{s,i}$	braking time of the i−th=1,2,3,4 friction material (s)
T	temperature (°C)
$T_{a}$	initial/ambient temperature (°C)
$W_{0}$	kinetic energy of the system (J)
$W_{i}$	work of friction of the i−th=1,2,3,4 friction material (J)
z	axial coordinate (m)
**Greek Symbols**	
ω	angular velocity ($\mathrm{rad}\;s^{-1}$)
$\omega_{0}$	initial angular velocity ($\mathrm{rad}\;s^{-1}$)

## References

[1] Yu L., Ma B., Chen M., Li H.Y., Liu J. (2020). Experimental study on the friction stability of paper-based clutches concerning groove patterns. Ind. Lubr. Tribol..

[2] Yu L., Ma B., Chen M., Li H., Ma C., Liu J. (2019). Comparison of the friction and wear characteristics between copper and paper based friction materials. Materials.

[3] Wang X., Ru H. (2019). Effect of Lubricating Phase on Microstructure and Properties of Cu–Fe Friction Materials. Materials.

[4] Liu J., Ma B., Li H., Chen M., Li G. (2018). Control strategy optimization for a dual-clutch transmission downshift with a single slipping clutch during the torque phase. Proc. Inst. Mech. Eng. Part D J. Automob. Eng..

[5] Marklund P., Larsson R. (2008). Wet clutch friction characteristics obtained from simplified pin on disc test. Tribol. Int..

[6] Biryukov V.P., Il’yushenko A.F., Leshok A.V., Pinchuk T.I. (2020). Influence of carbon-containing additives in the composition of copper-based friction materials on boundary friction in mineral and synthetic oils. J. Frict. Wear.

[7] Anderson A.E., Samal P.K., Newkirk J.W. (1984). Friction and wear of automotive brakes. ASM Handbook, Volume 7: Powder Metallurgy.

[8] Xie F., Hu W., Ning D., Zhuo L., Deng J., Lu Z. (2018). ZnO nanowires decoration on carbon fiber via hydrothermal synthesis for paper-based friction materials with improved friction and wear properties. Ceram. Int..

[9] Bijwe J., Majumdar N., Satapathy B.K. (2005). Influence of modified phenolic resins on the fade and recovery behavior of friction materials. Wear.

[10] Ilyushchanka A.P., Leshok A.V., Dyachkova L.N., Alekseenko N.A. Formation of the friction surface of a friction material based on copper depending on the amount of tin under lubrication condition. Proceedings of the 10th international scientific conference Balttrib’ 2019, Vytautas Magnus University.

[11] Prapai J., Morakotjinda M., Yotkaew T., Vetayanukul B. (2013). Tribological properties of PM Cu-based dry friction clutch. Key Eng. Mater..

[12] Tosangthum N., Krataitong R., Tongsri R. (2013). Sintered frictional materials based on Cu powders. Adv. Mat. Res..

[13] Liashok A., Dyachkova L., Feldshtein E., Jakubowski J., Patalas-Maliszewska J. (2020). On the effect of the grade and content of graphite on the structure, strength and tribological bechavior of friction materials based on tin bronze. Inżynieria Produkcji, Badania w Inżynierii Mechanicznej.

[14] Leshok A.V. Influence of graphite-containing additives of cast and shungite coke on tribotechnical properties of iron-based friction material. Proceedings of the International Scientific and Technical Conference “Polymer Composites and Tribology” “Polikomtrib-2019”.

[15] Abdullah O.I., Schlattmann J. (2013). Contact Analysis of a Dry Friction Clutch System. Int. Sch. Res. Not..

[16] Sarkar S., Sarswat P.K., Free M.L. (2018). Metal oxides and novel metallates coated stable engineered steel for corrosion resistance applications. Appl. Surf. Sci..

[17] Sarswat P.K., Sarkar S., Murali A., Huang W., Tan W., Free M.L. (2019). Additive manufactured new hybrid high entropy alloys derived from the AlCoFeNiSmTiVZr system. Appl. Surf. Sci..

[18] Zagrodzki P. (1985). Numerical analysis of temperature fields and thermal stresses in the friction discs of a multidisc wet clutch. Wear.

[19] Zagrodzki P. (1990). Analysis of thermomechanical phenomena in multidisc clutches and brakes. Wear.

[20] Zagrodzki P., Truncone S.A. (2003). Generation of hot spots in a wet multidisk clutch during short-term engagement. Wear.

[21] Della Gatta A., Iannelli L., Pisaturo M., Senatore A., Vasca F. (2018). A survey on modeling and engagement control for automotive dry clutch. Mechatronics.

[22] Awrejcewicz J., Grzelczyk D. (2013). Modeling and analysis of thermal processes in mechanical friction clutch—Numerical and experimental investigations. Int. J. Struct. Stab. Dy..

[23] Grzelczyk D., Awrejcewicz J. (2015). Wear Processes in a Mechanical Friction Clutch: Theoretical, Numerical, and Experimental Studies. Math. Probl. Eng..

[24] Abdullah O.I., Schlattmann J., Majeed M.H., Sabri L.A. (2018). The temperatures distributions of a single-disc clutches using heat partitioning and total heat generated approaches. Case Stud. Therm. Eng..

[25] Topczewska K., Schlattmann J., Abdullah O.I. (2020). Temperature and thermal stresses distributions in a dry friction clutch. J. Theor. Appl. Mech..

[26] Yevtushenko A.A., Grzes P. (2010). The FEM-modeling of the frictional heating phenomenon in the pad/disc tribosystem (a review). Numer. Heat Tr. A Appl..

[27] Yevtushenko A.A., Grzes P., Adamowicz A. (2015). Numerical analysis of thermal stresses in disk brakes and clutches (a review). Numer. Heat Tr. A Appl..

[28] Wasilewski P. (2020). Frictional heating in railway brakes: A review of numerical models. Arch. Computat. Methods Eng..

[29] Deressa K.T., Ambie D.A. (2021). Thermal load simulations in railway disc brake: A systematic review of modelling temperature, stress and fatigue. Arch. Computat. Methods Eng..

[30] Pisaturo M., Senatore A. (2016). Simulation of engagement control in automotive dry-clutch and temperature field analysis through finite element model. Appl. Therm. Eng..

[31] Wenbin L., Jianfeng H., Jie F., Liyun C., Chunyan Y. (2016). Simulation and application of temperature field of carbon fabric wet clutch during engagement based on finite element analysis. Int. Commun. Heat Mass.

[32] Biczó R., Kalácska G., Mankovits T. (2020). Micromechanical model and thermal properties of dry-friction hybrid polymer composite clutch facings. Materials.

[33] Yevtushenko A.A., Grzes P., Adamowicz A. (2020). The temperature mode of the carbon-carbon multi-disc brake in the view of the interrelations of its operating characteristics. Materials.

[34] Yevtushenko A.A., Grzes P. (2020). Initial selection of disc brake pads material based on the temperature mode. Materials.

[35] Agroskin A.A. (1980). Thermal Physics of Solid Fuel.

[36] Lutkov A.I. (1990). Thermal and Electrical Properties of Carbon Materials.

[37] Norley J., Tzeng J.J.-W., Getz G., Klug J., Fedor B. The development of a natural graphite heat-spreader. Proceedings of the Seventeenth Annual IEEE Semiconductor Thermal Measurement and Management Symposium.

[38] COMSOL Multiphysics® v. 5.3..

